# Effectiveness of anastomotic reinforcement sutures in reducing anastomotic leakage risk after laparoscopic rectal cancer surgery: a pooled and integration analysis

**DOI:** 10.3389/fonc.2024.1337870

**Published:** 2024-06-04

**Authors:** Yumin Yue, Xiaolong Zhang, Yaqi Qu, Xu Zhao, Fanghui Ding, Jiang Li, Bobo Zheng

**Affiliations:** ^1^ Department of General Surgery, Shaanxi Provincial People’s Hospital, Xi’an, China; ^2^ General Surgery Department, The First Hospital of Lanzhou University, Lanzhou, China; ^3^ National Cancer Center/National Clinical Research Center for Cancer/Cancer Hospital, Chinese Academy of Medical Sciences and Peking Union Medical College, Beijing, China

**Keywords:** anastomotic reinforcement sutures, anastomotic leakage, rectal cancer, laparoscopic surgery, pooled and integration analysis

## Abstract

**Background and objectives:**

Anastomotic leakage (AL) is one of the most serious complications after laparoscopic anus-preserving surgery for rectal cancer, which significantly prolongs the patient’s hospital stay, leads to dysfunction, and even increases the patient’s perioperative morbidity and mortality, and little is known about the effectiveness of anastomotic reinforcement sutures to prevent AL. Thus, this study was conducted to evaluate the efficacy of anastomotic reinforcement sutures as a means to prevent AL during laparoscopic surgery for rectal cancer.

**Methods:**

A comprehensive and systematic search was performed in the literature database by combining subject and free terms up to 10 October 2023. The overall literature included was integrated and analyzed using Stata 12.0 software and Review Manager version 5.4 software to assess the effect of anastomotic reinforcement sutures on the incidence of AL.

**Results:**

A total of 2,452 patients from 14 studies were included, and an integrated analysis showed that the use of anastomotic reinforcement sutures significantly reduced the incidence of AL [odds ratio (OR) = 0.26; 95% confidence interval (CI), 0.18–0.37; P < 0.00001; I^2 =^ 0%]. However, the findings confirmed whether or not the anastomosis reinforced with sutures did not affect the incidence of anastomotic stenosis (OR = 0.69; 95% CI, 0.37–1.32; P = 0.27; I^2^ = 0%). We performed subgroup analyses of the results of the study, the randomized controlled studies (OR = 0.31; 95% CI, 0.15–0.65; P < 0.001) as well as retrospective studies (OR = 0.28; 95% CI, 0.19–0.41; P < 0.001), 3–0 sutures (OR = 0.28; 95% CI, 0.17–0.45; P < 0.001) versus 4–0 sutures (OR = 0.26; 95% CI, 0.13–0.53; P < 0.001), barbed wire sutures (OR = 0.26; 95% CI, 0.14–0.48; P < 0.001) versus non-barbed wire sutures (OR = 0.30; 95% CI, 0.20–0.46; P < 0.001), interrupted (OR = 0.30, 95% CI, 0.20–0.46; P < 0.001) versus continuous sutures (OR = 0.29, 95% CI, 0.16–0.51; P < 0.001) to the anastomosis, full-thickness suture (OR = 0.29; 95% CI, 0.16–0.51; P < 0.001) versus sutured with the seromuscular layer (OR = 0.27; 95% CI, 0.14–0.53; P < 0.001), anastomotic sutured in one (OR = 0.27; 95% CI, 0.14–0.53; P < 0.001) versus non-one circle (OR = 0.30; 95% CI, 0.20–0.44; P < 0.001), and reinforcing sutures to the dog-ear area (OR = 0.26; 95% CI, 0.14–0.50; P < 0.001) versus the non–dog-ear area (OR = 0.30; 95% CI, 0.20–0.45; P < 0.001), which have suggested that there is no significant difference between each other and that all of them reduce the incidence of AL.

**Conclusions:**

This study provides evidence that performing reinforcement suturing of the anastomosis during laparoscopic rectal surgery significantly lowers the incidence of postoperative AL but has no significant effect on anastomotic stenosis. It is important to note that further randomized controlled studies are required to confirm this conclusion.

**Systematic review registration:**

https://www.crd.york.ac.uk/PROSPERO/, identifier CRD42022368631.

## Introduction

Anastomotic leakage (AL) is the most serious complication of laparoscopic surgery for rectal cancer with a high mortality rate (1). In recent years, with the continuous improvement of the stapler and manual suturing techniques, preoperative evaluation of patients, and surgical techniques for rectal anastomosis, the incidence of AL still falls within the range of 2.8%–30%. Out of these cases, 75% occurs in rectal anastomosis, resulting in a mortality rate of 2%–16.4% and a morbidity rate of 20%–35% (2–4).

It has been shown that the risk factors for the occurrence of anastomotic leaks are closely related to the level of anastomosis, anastomotic blood supply, anastomotic tension, anastomotic technique, and the degree of anastomotic reinforcement (5, 6). Previous studies have reported that, in laparoscopic anterior rectal resection, the straight line formed by the linear cutting closure to dissect the rectum and the circular line of the tubular anastomosis inevitably form two intersecting corners (“dog-ear zone”). This brings about a high incidence of AL due to the structural weakness and lack of blood supply of the intersecting part of the pegs and is susceptible to inflammation and edema (7, 8). Therefore, surgeons tend to reinforce the anastomotic site, especially the “dog-ear” area, to minimize postoperative AL. However, there is still some controversy regarding the effectiveness of anastomotic reinforcement sutures in improving AL. Placer et al. (9), in a prospective randomized controlled study, showed that bioabsorbable suture reinforcement of colorectal anastomoses more than 5 cm away from the anal verge did not reduce the incidence of anastomotic complications (i.e., leakage, bleeding, or stenosis). Similarly, Senagore et al. (10) have concluded that reinforcement of colorectal anastomoses with bioabsorbable material did not affect the incidence of anastomotic leaks but may reduce anastomotic strictures. However, Lin et al. (11), in a study of anastomotic reinforcement with barbed sutures during laparoscopic low anterior resection of rectal cancer, found that the incidence of AL was 10% in the control group and 2.82% in the suture-reinforced group, suggesting that reinforcement of the anastomotic “dog’s ear” area with sutures is associated with a low incidence of AL. Similarly, Ban et al. (12) have demonstrated that reinforcement of the anastomosis with barbed sutures after laparoscopic rectal cancer resection significantly reduced the incidence of AL. Several retrospective clinical studies have confirmed that reinforcement suturing of the anastomosis reduces the incidence of AL. However, to date, few studies have compared the efficacy assessment of patients who underwent anastomotic reinforcement with those who did not. There is still some controversy as to whether anastomotic reinforcement suturing reduces the incidence of AL.

Our study aimed to evaluate if reinforcing anastomotic sutures during laparoscopic rectal resection reduces the incidence of AL. In this study, we analyzed the clinical studies published in recent years on reinforced versus unreinforced sutures in laparoscopic surgery for rectal resection. Moreover, this study provides guidance and evidence-based medicine for the prevention of AL in laparoscopic surgery for rectal resection.

## Materials and methods

This pooled analysis was based on the Preferred Reporting Items for Systematic Reviews and Meta-Analysis (PRISMA) guidance (13). The study protocol was registered with the International Prospective Register of Systematic Reviews (PROSPERO registration number: CRD42022368631).

### Literature search strategy

Literature searches were conducted by two independent authors in PubMed, Embase, Cochrane Library, Web of Knowledge, and Clinical Trials.gov databases for medical subject terms and free words. From the time of database inception until 20 October 2023, the last database search was updated on 10 November 2022. There were no language restrictions. The search terms that we used were as follows: laparoscopic surgery for rectal cancer, anastomosis, AL, and reinforcement. The search strategy on PubMed is as follows: (Anastomotic) AND (((laparoscopic surgery) AND (rectal)) AND (Reinforcement)).

### Inclusion and exclusion criteria

The clinical studies included in this integrative analysis met the following criteria: (1) All patients included in the study were required to undergo laparoscopic rectal resection. (2) The intervention group used reinforcing sutures to close the anastomosis without limiting the type of reinforcing sutures and the method of reinforcing sutures, and the control group did not use reinforcing sutures.

Exclusion criteria are as follows: single-arm clinical studies, observational studies, abstracts, as well as reviews.

### Data acquisition

Two researchers individually screened the titles or abstracts of the retrieved literature and, when necessary, the full text of the articles. They also reviewed the included literature with each other for accuracy and completeness of data, and a third researcher participated in or jointly discussed any disagreements that arose during the process. When an article does not contain information on a specific endpoint, an attempt was made to contact the authors to clarify details and/or request missing outcome data.

### Outcomes

The primary endpoint was the occurrence of AL and the secondary endpoint was the occurrence of anastomotic stenosis.

### Quality evaluation

Two investigators independently assessed the risk of bias using the latest version of the Cochrane Collaboration tool to guide the assessment of randomized controlled studies (14). The quality of the literature was rated as low, high, or unclear, and the results of the risk of bias assessment were used only to describe the quality of the literature and were not used as criteria for study selection. The quality of retrospective studies was assessed using the Newcastle-Ottawa scale (NOS) (15), which includes mainly the selection of study subjects, comparability between groups, and outcome or exposure assessment. A full score of 9 was assigned, where 1–3 was considered as low quality, 4–6 as moderate quality, and 7–9 as high quality.

### Statistical analysis

Endpoints were expressed as odds ratios (OR) for dichotomous data and with 95% confidence interval (CI). Heterogeneity between studies was assessed with the I² statistic; when I² = 0, there was no heterogeneity; the larger the I² statistic, the greater the heterogeneity (I² < 25%, 25%–50%, and >50% indicate low, moderate, and high heterogeneity, respectively), and if I² > 50% indicates the presence of more pronounced heterogeneity, then the analysis was performed using a random-effects model, and vice versa, using a fixed-effects model. When heterogeneity was found in the main results, a separate sensitivity analysis was performed to find potential sources of heterogeneity. We used the software RevMan 5.4 and Stata 12.0 to perform statistical analyses. In addition, publication bias was assessed using funnel plots. The only way to control for publication bias is to collect as comprehensively as possible all studies that meet the inclusion criteria.

A subgroup analysis of the effect of AL was performed by the type of study in the literature (retrospective and randomized controlled studies), 3–0 versus 4–0 suture, barbed versus non-barbed suture, interrupted versus continuous suture, full-thickness versus seromuscular suture, one-circle versus non–one-circle suture, and closure of the dog’s ear versus non-dog’s ear.

## Results

### Study details

**Figure 1:**
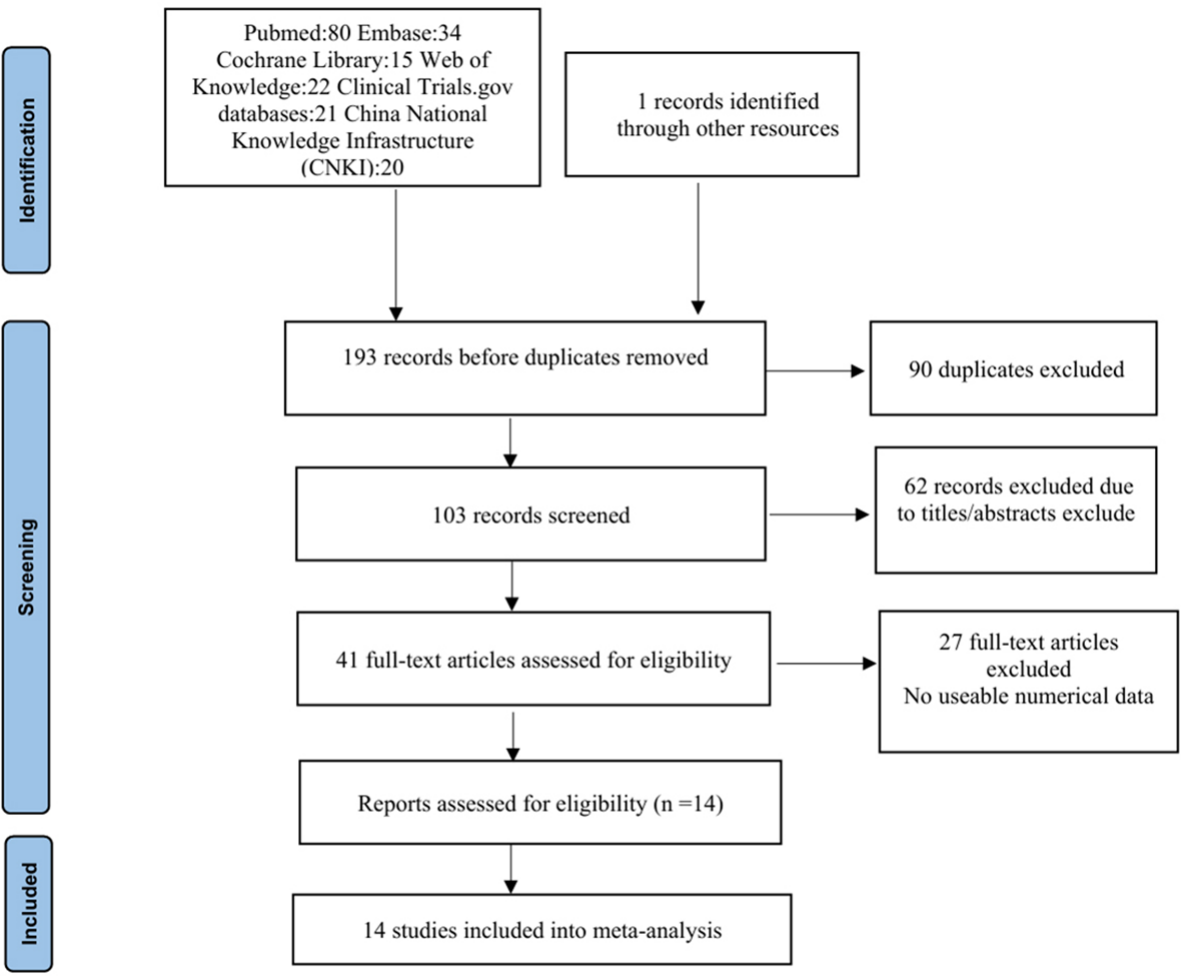
PRISMA diagram showing identification of studies from the initial literature search.

Based on the search strategy, 193 relevant studies were initially identified. After removing 90 duplicates, a total of 103 potential studies were included. Sixty-two studies were excluded in the title and abstract screening, and 41 studies were considered eligible for full-text assessment. In addition, 27 full-text were excluded, 19 studies were excluded because they were single-arm studies, and eight studies did not provide data information because they were clinical guidelines. Fourteen studies met the inclusion criteria for this analysis (12 retrospective studies (11, 12, 16–25) versus two randomized controlled studies (26, 27)). A total of 14 studies (11, 12, 16–27) with 2,452 patients (reinforced suture group, n = 1196, and non-reinforced suture group, n = 1,256) were included. All patients included in the study underwent rectal resection. The flow chart of this study is shown in **Figure 1**.

### Study of baseline information

**Tables 1**, **2** incorporate study details from 14 studies, which include patient demographics, procedure-related details, postoperative complications, and the type of suture used during anastomotic reinforcement suturing.

**Table 1:** Characteristics of patients included in the study.

	N (RS/NRS)		Male (RS/NRS, n)	Age (year)		BMI (kg/m^2^)		Tumor diameter (cm)	
				RS	NRS	RS	NRS	RS	NRS
Baek, 2013 (23)	47/63		29/31	64.1 ± 9.8	61.4 ± 11.0	24.1 ± 3.1	23.5 ± 2.7	NR	NR
Chen, 2014 (22)	56/64		38/38	≥60:31	≥60:40	NR	NR	≥5:21	≥5:28
Maeda, 2015 (16)	91/110		52/66	<75:67	<75:86	<25:74	<25:81	<4:71	<4:87
He, 2018 (26)	145/146		78/85	<70:109	<70:114	<24:117	<24:111	<4:116	<4:115
Zhang, 2018 (24)	60/60		31/29	53.67 ± 14.22	55.18 ± 13.78	22.35 ± 2.81	21.79 ± 3.11	NR	NR
Luo, 2020 (21)	86/129		51/71	62.8 ± 1.0	60.7 ± 1.0	22.1 ± 0.3	21.9 ± 0.3	4.3 ± 0.2	4.6 ± 0.2
Zhang, 2021 (20)	26/32		19/22	49.3 ± 1.5	49.3 ± 1.5	NR	NR	2.9 ± 0.2	3.1 ± 0.1
Jiang, 2021 (18)	82/42		53/23	61.35 ± 12.33	61.6 ± 11.4	23.37 ± 2.85	22.35 ± 2.95	NR	NR
Ban, 2022 (12)	168/151		80/73	61.8 ± 8.7	63.0 ± 9.7	23.2 ± 3.6	22.8 ± 3.8	4.4 ± 1.7	4.1 ± 1.8
Hashida, 2022 (17)	72/81		45/45	68.1	68.6	22.9	23	3.8	3.5
Jin, 2022 (19)	123/135		84/75	61.95 ± 11.62	61.81 ± 13.46	23.40 ± 3.02	23.26 ± 4.71	3.76 ± 1.66	3.77 ± 1.42
Yang, 2022 (25)	38/38		20/20	52.19 ± 6.20	52.16 ± 6.22	NR	NR	3.64 ± 0.42	3.44 ± 0.46
Lin, 2022 (11)	142/150		79/78	65.00 (7.53)	59.50 (10.57)	22.28 (2.85)	21.41 (2.89)	3.75 (1.48)	4.00 (1.57)
Zhang, 2023 (27)	198/205		115/122	66 (58.25–71)	65 (57–70)	24 (22.33– 25.78)	23.6 (21.7–25.3)	3.50 [2.52, 4.50]	3.50 [3.00, 4.50]

	Ligation of left colic artery			Tumor site (from the anal verge)		Surgical time (min)		Estimated blood loss (mL)	
	RS		NRS	RS	NRS	RS	NRS	RS	NRS
Baek, 2013 (23)	NR		NR	9.7 ± 3.9	9.7 ± 3.6	198.3 ± 75.7	212.1 ± 65.0	174.5 ± 348.0	188.4 ± 301.5
Chen, 2014 (22)	NR		NR	≥5:15	≥5:18	211 ± 91	174 ± 57	119 ± 38	121 ± 46
Maeda, 2015 (16)	48		70	≥5:66	≥5:90	<240:52	<240:61	<60:75	<60:85
He, 2018 (26)	22		26	≥5:112	≥5:118	≥180:69	≥180:62	<50:128	<50:124
Zhang, 2018 (24)	NR		NR	7.54 ± 2.12	6.95 ± 1.87	158.62 ± 30.17	150.02 ± 28.95	54.75 ± 10.48	56.81 ± 9.96
Luo, 2020 (21)	NR		NR	NR	NR	160.2 ± 3.8	128.9 ± 2.4	127 ± 9	114 ± 6
Zhang, 2021 (20)	NR		NR	4.0 ± 0.2	4.1 ± 0.1	143.4 ± 2.5	118.4 ± 2.0	91.4 ± 7.9	77.5 ± 6.3
Jiang, 2021 (18)	NR		NR	NR	NR	109.5 ± 36.38	103.8 ± 26.2	83.1 ± 52.10	97.0 ± 47.9
Ban, 2022 (12)	79		75	≥5: 123 (73.2)	≥5: 114(75.5)	150.4 ± 25.1	146.6 ± 20.2	60.5 ± 43.9	58.2 ± 46.3
Hashida, 2022 (17)	54		61	6.2	6.8	301	285	5.6	9.7
Jin, 2022 (19)	NR		NR	9.25 ± 2.69	8.53 ± 3.31	124.66 ± 25.27	116.73 ± 38.07	48.77 ± 18.87	52.15 ± 22.26
Yang, 2022 (25)	NR		NR	3.56 ± 0.15	3.61 ± 0.13	160.23 ± 3.85	128.95 ± 2.46	114.90 ± 9.85	119.54 ± 6.27
Lin, 2022 (11)	NR		NR	7.00 (1.18)	7.00 (1.27)	147.50 (35.05)	130.00 (32.07)	100.00 (73.96)	100.00 (75.01)
Zhang, 2023 (27)	NR		NR	10 (8–12)	8 (6–10)	105 (92.75–124)	100 (81–120)	60 (50–60)	60 (50–60)

NR, not reported; RS, reinforced suture; NRS, unreinforced suture.

**Table 2:** Detailed details of anastomosis reinforcement.

Study, year	Type of reinforcement suture	The suture level was reinforced	Type of anastomosis reinforcement	Reinforcement area
Baek, 2013 (23)	Circular stapler (CDH 29 mm, Ethicon)	Full-layer suture	Interrupted sutures	Two corners made by crossing the circular and linear staple lines
Chen, 2014 (22)	3–0 absorbable suture	Seromuscular layer suture	Not reported	The “end-angle” anastomosis
Maeda, 2015 (16)	4–0 PDS (Ethicon Inc., New Jersey, USA)	Not reported	Interrupted sutures	The two corners that crossed the circular and linear staple lines were always included.
He, 2018 (26)	4–0 Absorbable suture	Not reported	Interrupted sutures	“Dog ears” area
Zhang, 2018 (24)	4–0 Absorbable sutures	Not reported	Interrupted sutures	The lateral side of the anastomosis
Luo, 2020 (21)	a barbed suture	Seromuscular layer suture	Continuous suture to close the gap	“Dog ears” area
Zhang, 2021 (20)	3–0 Vicryl thread	Full-layer suture	Continuous suture to close the gap	Around the entire circular anastomosis
Jiang, 2021 (18)	3-0 Absorbable suture	Seromuscular layer suture	Interrupted sutures	Placement of a “**Figure 2**” suture on either both or one side of the anastomosis
Ban, 2022 (12)	3-0 V-Loc 180 sutures (Covidien, Mansfield, MA, United States)	Full-layer suture	Continuous suture to close the gap	Reinforce the intersection of the cutting lines and anterior anastomosis wall
Hashida 2022 (17)	3–0 PDS (Ethicon Inc., New Jersey, USA)	Not reported	Interrupted sutures	Two corners made by intersecting a circular staple line and a straight staple line
Jin, 2022 (19)	3–0 Barbed suture	Full-layer suture	Not reported	Around the entire circular anastomosis
Yang, 2022 (25)	A barbed suture	Seromuscular layer suture	Continuous suture to close the gap	“Dog ears” area
Lin, 2022 (11)	V-LOC™ barbed suture (3–0) (COVIDIEN, Beijing, China)	Seromuscular layer suture	Continuous suture to close the gap	“Dog ears” area
Zhang, 2023 (27)	A barbed suture	Full-layer suture	Not reported	around the entire circular anastomosis

**Figure 2:**
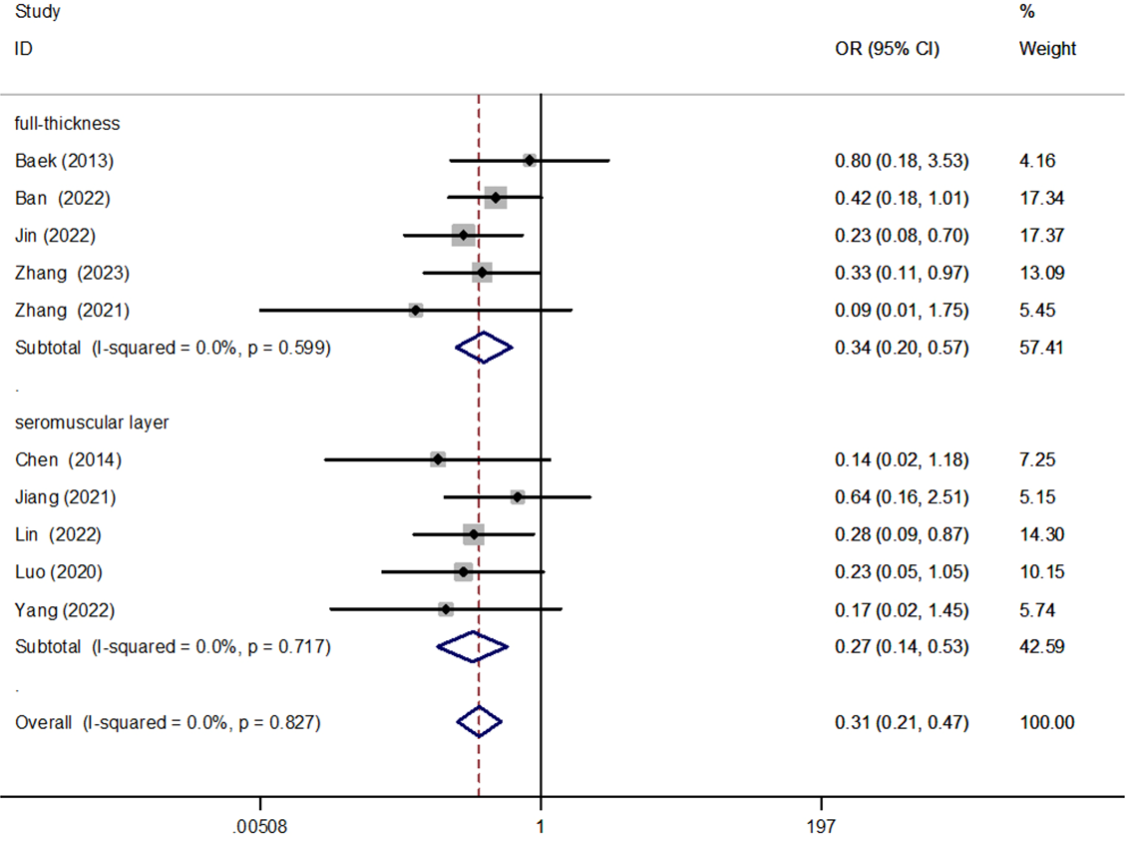
Forest plot of full-thickness versus seromuscular suture in the subgroup analysis of AL incidence.

### Literature quality evaluation

The quality of the literature for randomized controlled studies was evaluated. All two randomized controlled studies were grouped using either the random number method or the sealed envelope random assignment method, and one study was blinded. All two studies reported complete data for each outcome indicator, but other sources of bias were not clear. All 12 retrospective cohort studies that were included in this analysis were rated as high quality. Out of the 12 studies, five had a score of 7, four had a score of 8, and three had a score of 9 out of a possible 9 on the NOS. This indicates a high level of quality for the included studies, with NOS scores mostly ranging between 7 and 9 (**Table 3**). The large number of high-quality studies used in this analysis helps to reduce bias and increase the credibility of the results. Therefore, the high quality of the studies included in this analysis makes the resulting meta-analysis more credible.

**Table 3:** Quality assessment of studies included in the final analysis according to the Newcastle-Ottawa scale (NOS).

Study, year	Selection	Comparability	Exposure/outcome	Total	Overall quality
Baek, 2013 (23)	3	1	3	7	High
Chen, 2014 (22)	3	2	2	7	High
Maeda, 2015 (16)	3	1	3	7	High
Zhang, 2018 (24)	3	1	3	7	High
Luo, 2020 (21)	3	1	3	7	High
Zhang, 2021 (20)	4	2	2	8	High
Jiang, 2021 (18)	4	1	3	8	High
Ban, 2022 (12)	4	2	3	9	High
Hashida, 2022 (17)	4	2	2	8	High
Jin, 2022 (19)	4	2	3	9	High
Yang, 2022 (25)	4	2	3	9	High
Lin, 2022 (11)	4	2	2	8	High

### Primary outcomes

#### AL

**Figure 3:**
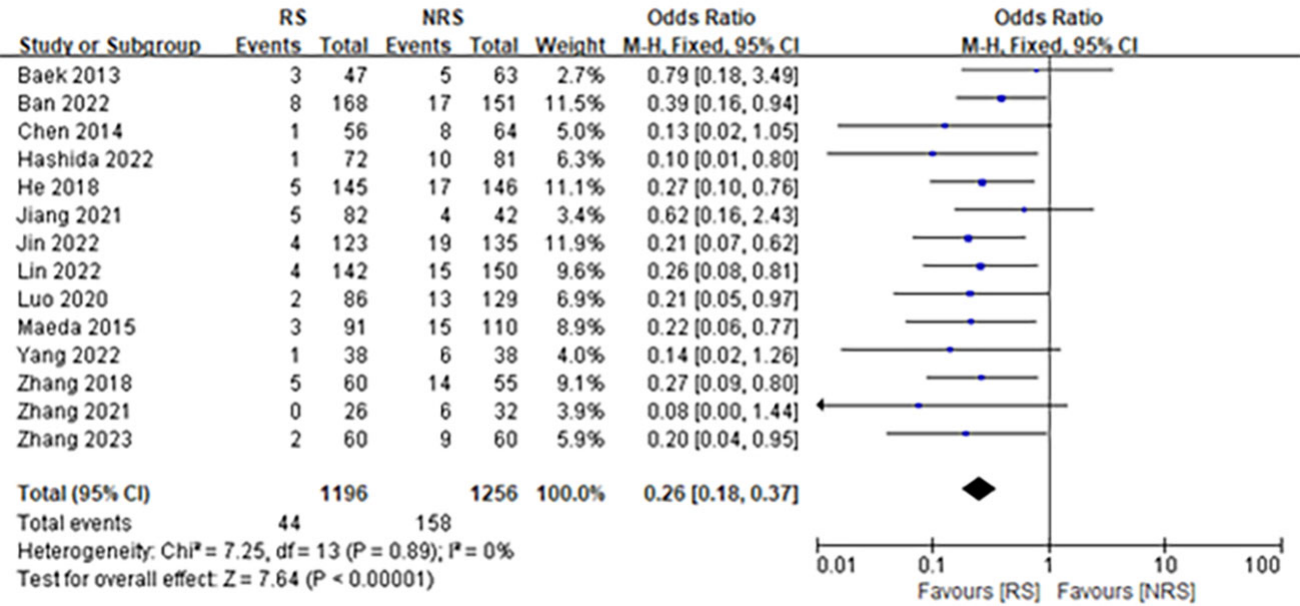
Forest plot comparing the incidence rate of AL in patients with anastomotic reinforcement suture (RS) versus non-reinforcement suture (NRS).

Fourteen studies (11, 12, 16–27), which included 2,452 participants, were reported with data on AL after laparoscopic surgery for rectal resection. Pooled analysis showed that reinforced sutures to the anastomosis significantly reduced the risk of AL in patients undergoing laparoscopic surgery for rectal resection (OR = 0.26; 95% CI, 0.18–0.37; P < 0.00001; I^2^ = 0%; **Figure 3**).

#### Anastomotic stenosis

**Figure 4:**
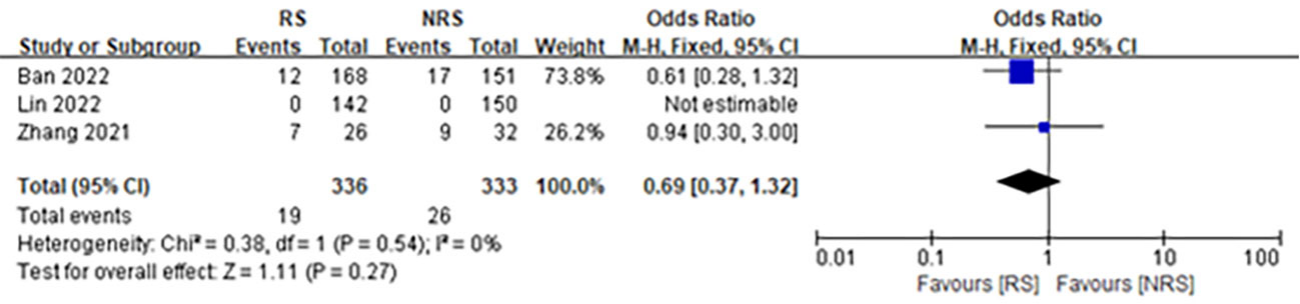
Forest plot comparing the incidence of anastomotic stenosis in patients with reinforced (RS) versus non-reinforced anastomotic sutures (NRS).

There were three studies (11, 12, 20), consisting of 669 participants, reporting data on anastomotic stenosis after laparoscopic surgery for rectal resection. The results revealed that anastomotic reinforcement sutures did not significantly affect anastomotic stenosis following laparoscopic surgery for rectal resection (OR = 0.69; 95% CI, 0.37–1.32; P = 0.27; I^2^ = 0%) (**Figure 4**).

### Subgroup analysis

#### Retrospective and randomized

**Figure 5:**
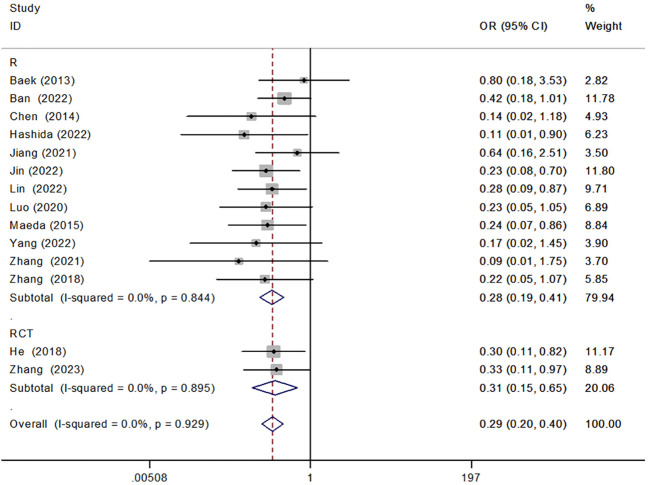
Forest plot of retrospective versus randomized controlled studies in the subgroup analysis of AL incidence.

We analyzed the results of both retrospective [OR = 0.28, 95% CI (0.19, 0.41), P < 0.001] and randomized [OR = 0.31, 95% CI (0.15, 0.65), P < 0.001] controlled studies according to the type of study, showing that reinforced sutures to the anastomosis significantly reduced the incidence of AL (**Figure 5**).

#### 3–0 and 4–0 sutures

**Figure 6:**
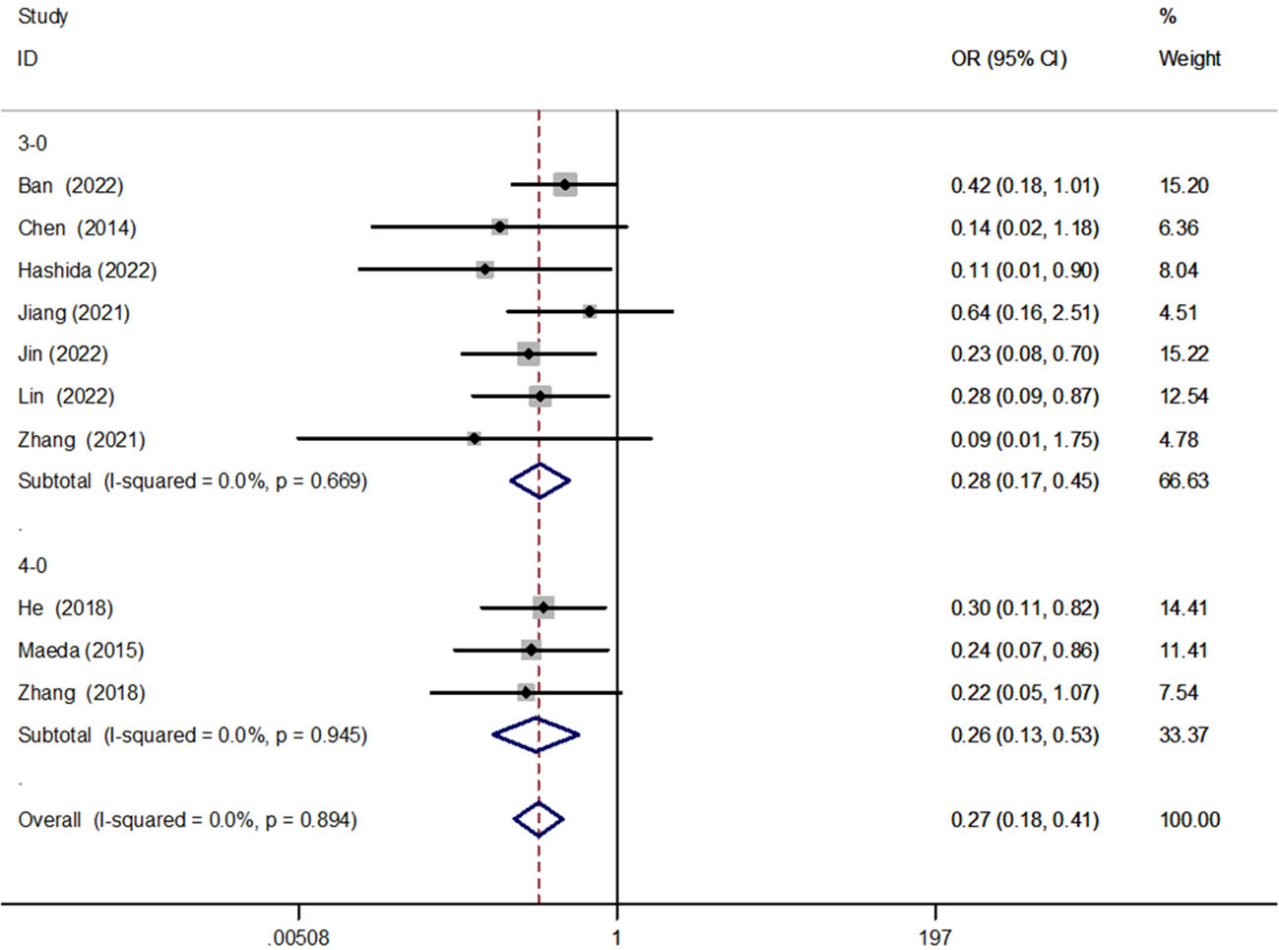
Forest plot of 3–0 versus 4–0 suture in the subgroup analysis of AL incidence.

We performed a subgroup analysis of anastomotic reinforcement sutures using 3–0 versus 4–0 sutures, and the results showed that both anastomotic reinforcement sutures using 3–0 [OR = 0.28, 95% CI (0.17, 0.45), P < 0.001] versus 4–0 [OR = 0.26, 95% CI (0.13, 0.53), P < 0.001] sutures reduced the incidence of AL, and there was no significant difference between the two (**Figure 6**).

#### Barbed sutures and non-barbed sutures

**Figure 7:**
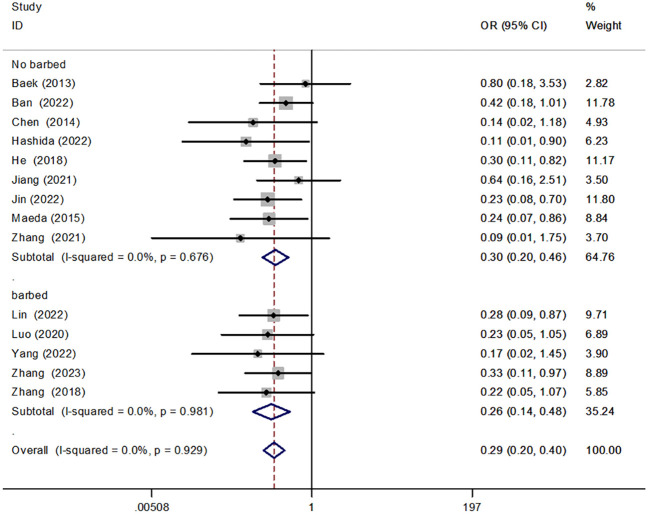
Forest plot of barbed versus non-barbed suture in the subgroup analysis of AL incidence.

We conducted a subgroup analysis to compare the use of barbed versus non-barbed sutures in anastomotic reinforcement sutures. The results indicated that anastomotic reinforcement sutures using barbed sutures [OR = 0.26, 95% CI (0.14, 0.48), P < 0.001] reduced the incidence of AL when compared to non-barbed sutures [OR = 0.30, 95% CI (0.20, 0.46), P < 0.001]. However, there was no significant difference between the two approaches (**Figure 7**).

#### Interrupted suture and continuous suture

**Figure 8:**
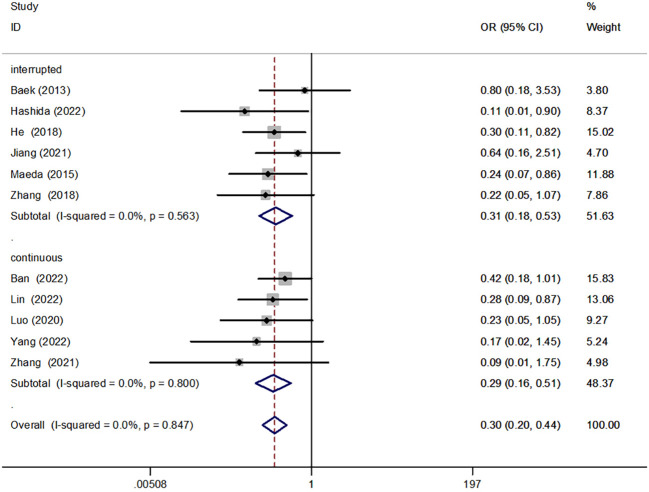
Forest plot of interrupted versus continuous suture in the subgroup analysis of AL incidence.

We conducted a subgroup analysis comparing interrupted [OR = 0.31, 95% CI (0.18, 0.53), P < 0.001] and continuous sutures [OR = 0.29, 95% CI (0.16, 0.51), P < 0.001] for anastomotic reinforcement. Results indicated that both suture techniques reduced the incidence of AL (**Figure 8**).

#### The full-thickness suture was sutured with the seromuscular layer

We performed a subgroup analysis of full-thickness suture and seromuscular suture for anastomotic reinforcement. The results showed that both full-thickness sutures [OR = 0.34, 95% CI (0.20, 0.57), P < 0.001] and seromuscular suture [OR = 0.27, 95% CI (0.14, 0.53), P < 0.001] could reduce the incidence of AL, but there was no significant difference between them (**Figure 2**).

#### The anastomosis was sutured in one and non-one circle

**Figure 9:**
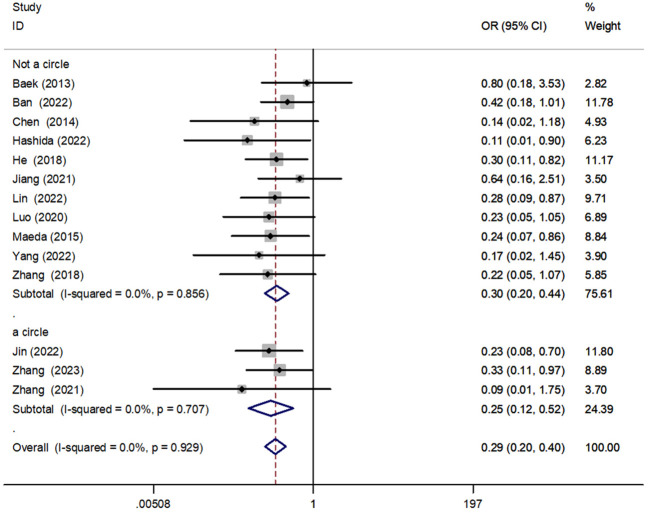
Forest plot of one-circle versus non–one-circle suture in the subgroup analysis of AL incidence.

We performed a subgroup analysis of anastomotic reinforcement sutures for anastomotic one-circle versus non–one-circle sutures. The results showed that both one-circle [OR = 0.25, 95% CI (0.12, 0.52), P < 0.001] versus non–one-circle sutures [OR = 0.30, 95% CI (0.20, 0.44), P < 0.001] for anastomotic reinforcement reduced the incidence of AL, with no significant difference between the two (**Figure 9**).

#### The dog-ear area was sutured to the non–dog-ear area

**Figure 10:**
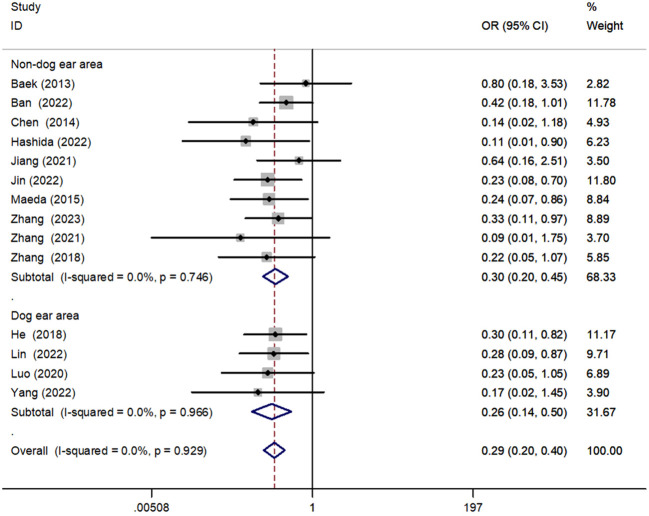
Forest plot of closure of the dog’s ear versus the non–dog’s ear in the subgroup analysis of AL incidence.

We performed a subgroup analysis of anastomotic reinforcement sutures for the dog-ear region versus the non–dog-ear region. The results showed that the incidence of AL was reduced for both dog-ear [OR = 0.26, 95% CI (0.14, 0.50), P < 0.001] and non–dog-ear [OR = 0.30, 95% CI (0.20, 0.45), P < 0.001] regions, with no significant difference between the two (**Figure 10**).

#### Publication bias

**Figure 11:**
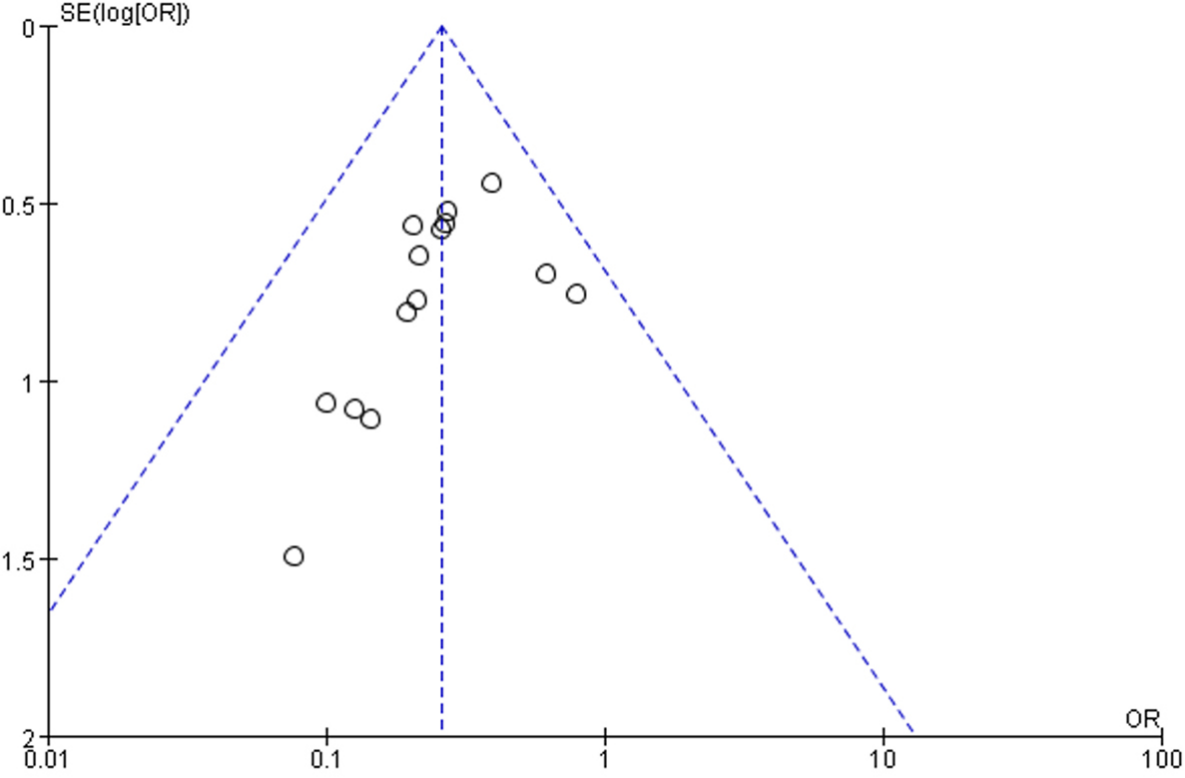
Funnel diagram of AL.

Analysis of publication bias is based on a funnel plot drawn to assess the effect of anastomotic reinforcement sutures on AL in patients undergoing rectal resection. This analysis revealed that the scatter of included studies was more evenly distributed, suggesting that the publication bias was not significant and that the results of this systematic analysis were reliable (**Figure 11**).

## Discussion

Our recent pooled analysis has confirmed that reinforcing the anastomotic suture during laparoscopic surgery for rectal cancer can significantly reduce the incidence of AL in patients who undergo laparoscopic surgery for rectal cancer. This study is the largest pooled and integration analysis of its kind, and its more reliable conclusions suggest that anastomotic reinforcement sutures significantly reduce AL.

AL is one of the most serious complications after laparoscopic surgery for rectal cancer, which induces intra-abdominal infection and pelvic abscess and seriously affects the patient’s postoperative quality of life and prognosis (28). The direct causes of AL include the blood supply of the anastomosis, the level of anastomosis technique, anastomotic tension, and intestinal luminal pressure. The current studies have confirmed that tumor size, distance from the tumor to the anal verge, and the use of reinforcement sutures to the anastomosis are independent risk factors for symptomatic AL (29). Lin et al. (11) conducted a retrospective study of 292 patients undergoing laparoscopic low anterior resection for rectal cancer. They found that the use of barbed sutures for anastomotic reinforcement was associated with a low incidence of AL. These results further suggest that the incidence of postoperative AL was 2.82% and 10%, respectively. These results further suggest that AL can be significantly reduced by reinforcing the anastomotic suture, strengthening the “dog-ear” region, and closing the rectal mesenteric space. As mentioned in our background section, the dog-ear region is a potential ischemic area for AL as the two suture angles of the rectal stump are created by rectal transection. In addition, when performing colorectal end-to-end anastomosis in rectal cancer surgery, a gap between the colonic and rectal mesentery tends to form, leading to delayed postoperative healing and increasing the risk of AL. Several studies have demonstrated that strengthening the “dog’s ear” area and closing the gap with sutures reduces anastomotic tension improve local blood supply and prevent anastomotic dehiscence (24, 26). Few previous studies have focused on the role of anastomotic reinforcement sutures in reducing the incidence of AL after laparoscopic surgery for rectal cancer. Earlier, Gadiot et al. (30) compared 76 cases that received attraction sutures with 77 cases that did not and found a significant decrease in the incidence of AL in the sutured group. Similarly, Maeda et al. (16). conducted a retrospective study on rectal cancer patients who underwent laparoscopic surgery. The study found that the distance between the tumor and the anal verge, the tumor size, and the use of reinforcing sutures were all independent risk factors for AL. The patients in this study were divided into two groups, a low-risk group (patients without any risk factors) and a high-risk group (patients with one or two risk factors), based on the site and size of their tumor. Among the high-risk group, patients who received reinforcing sutures had a significantly lower incidence of AL compared to patients who did not receive reinforcing sutures. However, no significant difference was observed in the low-risk group. In a study of laparoscopic endoluminal reinforcement sutures in laparoscopic rectal surgery, Hashida et al. (17). found that laparoscopic endoluminal reinforcement sutures significantly reduced the incidence of AL, whereas, in multivariate analysis, distance to the anal verge less than 6.5 cm, diabetes mellitus, and non-use of reinforcement sutures were shown to be the independent risk factors for AL.

Currently, most laparoscopic radical rectal cancer surgeries are performed with a double anastomosis device anastomosis, and clinical reports on further reducing AL through technical improvements are rare. In this study, we included all the clinical studies on the postoperative effects of anastomotic reinforcement sutures on patients after laparoscopic surgery for rectal resection, and the results confirmed that anastomotic reinforcement suture can significantly reduce the incidence of AL after laparoscopic surgery for rectal resection, and, therefore, we believe that anastomotic reinforcement suture is a clinical problem that surgeons need to pay great attention to and has a significant effect on reducing the incidence of postoperative complications after laparoscopic surgery for rectal resection. The incidence of complications after laparoscopic surgery for rectal resection is of great significance.

In this study, we conducted a pooled analysis of currently available randomized controlled and retrospective studies on anastomotic reinforcement suturing, 3–0 versus 4–0 suture, barbed versus non-barbed suture, interrupted versus continuous suture, full-thickness versus seromuscular suture, one-circle versus non–one-circle suture, and closure of the dog’s ear versus non–dog’s ear to further confirm that anastomotic reinforcement suturing significantly reduces the incidence of AL, which was similarly confirmed by subgroup analyses showing the results of randomized controlled studies. We also found that reinforcement suturing of the anastomosis could reduce the risk of anastomotic stenosis, but, due to the small number of included studies, the results may have some errors, so further validation is still needed in subsequent studies. Meanwhile, most of the studies included in this study were retrospective clinical studies with small sample sizes, and the methods of anastomotic reinforcement suturing varied from study to study, so there is a certain degree of variability, and we look forward to future multicenter prospective randomized controlled trials with large sample sizes to further validate the results. We have used this study to reinforce the anastomosis with sutures during laparoscopic rectal surgery.

## Conclusions

Through our integrated analysis study, we have confirmed that reinforcing sutures in the anastomosis significantly reduces the incidence of AL. This finding can provide useful guidance for clinical surgeons and has great value in reducing postoperative complications after rectal surgery. However, the present study requires a large number of randomized controlled studies to further confirm the reliability of this finding.

## Data availability statement

The original contributions presented in the study are included in the article/**Supplementary Material**. Further inquiries can be directed to the corresponding author.

## Author contributions

YY: Conceptualization, Data curation, Formal analysis, Software, Validation, Writing – original draft, Writing – review & editing. XLZ: Conceptualization, Data curation, Formal analysis, Software, Supervision, Writing – review & editing. YQ: Conceptualization, Data curation, Methodology, Project administration, Validation, Writing – review & editing. XZ: Formal analysis, Project administration, Software, Supervision, Validation, Writing – review & editing. FD: Conceptualization, Data curation, Formal analysis, Project administration, Resources, Validation, Visualization, Writing – review & editing. JL: Project administration, Resources, Supervision, Validation, Visualization, Writing – review & editing. BZ: Conceptualization, Data curation, Funding acquisition, Investigation, Methodology, Resources, Supervision, Validation, Visualization, Writing – review & editing.
